# Effects of training on attitudes of psychiatric personnel towards patients who self‐injure

**DOI:** 10.1002/nop2.45

**Published:** 2016-02-17

**Authors:** Vojna Tapola, Jarl Wahlström, Raimo Lappalainen

**Affiliations:** ^1^ Department of PsychologyUniversity of JyväskyläP.O. 35FI‐40014JyväskyläFinland

**Keywords:** Attitudes, evidence‐based practice, personnel, psychiatric, self‐injury, training, USP

## Abstract

**Background:**

Improving attitudes of personnel towards self‐injurious patients leads to better working alliance and contributes to better patient outcomes. Previous research into the improvement of these attitudes has recorded the need for specific training in evidence‐based assessment and treatment of self‐injurious patients.

**Aim:**

The current study describes the attitudes towards self‐injurious patients among psychiatric personnel. The study also evaluates the effect of a structured clinical training program on psychiatric personnel's attitudes towards patients who self‐injure. It further examines whether age, education, frequency of self‐injurious patients contact, and work experience of the personnel are associated with the existing attitudes.

**Methods:**

Psychiatric personnel (*N *=* *50) attended a four‐day training program, presenting evidence‐based knowledge regarding self‐injury assessment and treatment, using group exercises and reflective learning principles. The personnel completed the Understanding Suicidal Patients Questionnaire (USP) anonymously PreTraining, on 17 January 2014, and PostTraining, on 20 June 2014. The mean differences as well as single USP items before and after the training were tested by unpaired *t*‐test**.** Two‐way ANOVA was used to test impact of background variables on the USP scores.

**Results:**

The training program had statistically significant impact (*P* < 0·01) on the following individual items of the USP scale: Patients who have tried to commit suicide are usually treated well in my work unit (*d* = 1·02); A person who has made several suicide attempt is at greater risk of committing suicide (*d* = 0·64); Because the patients who have tried to commit suicide have emotional problems, they need the best possible treatment (*d* = 0·57). The results also suggested that the frequency of patient contact had impact on attitudes towards self‐injurious patients.

## Introduction

Suicide and self‐injurious behavior (SIB) are substantial health problems worldwide. It has been estimated that over 800 000 people die due to suicide every year and for each adult who died of suicide there may have been more than 20 others attempting suicide (World Health Organization 2014).

In Finland in 2012, the suicide rate per 100,000 people was 16·1 (OSF 2014). Suicide attempts are estimated to be 10‐20 times more frequent. The official diagnostic system in Finland is the WHO's classification system for diseases, the ICD‐10, (http://www.who.int/classifications/icd/en/) in which codes X69 and X70 to X84 classify attempted or completed suicide according to the way self‐injury has been inflicted. The same diagnoses are used to classify other forms of self‐injury where the result is not fatal. In other words, in Finland it is not possible to separate non‐suicidal self‐injurious behavior (NSSI) from suicide attempts. Because the two behaviors are equated in practice, in this paper the term SIB is used to cover all self‐injurious acts, irrespectively of suicide intent.

In Finland, the indirect financial cost defined as the value of lost production due to premature mortality as a result of suicide among working population, is estimated to be several hundreds of million euros per year (OECD 2014). The traumatic effects of both suicide and non‐fatal self‐injurious behavior further burden the friends and relatives, as well as health care staff involved in care of people who engage in self‐injurious behavior.

Previous research has suggested suicide prevention to depend on the attitude of health professionals; improving attitudes and understanding of self‐injurious patients should lead to better working alliance and contribute to better patient outcomes (Herron *et al*. 2001). Previous research on improving attitudes of personnel towards self‐injurious patients has indicated that there is a need for training (Crawford *et al*. 2003, McCann *et al*. 2007, Conlon & O'Tuathail 2012, Artis & Smith 2013). Improvements in attitudes following brief training in intervention programs have been noted (Appleby & Green 2000, Leung & Chan 2000, Samuelsson & Åsberg 2002). In their review of studies on nurses’ attitudes towards self‐injury Karman *et al*. (2015) further specify that the type of education resulting in improvement of attitudes towards patients who engage in SIB is that which contains reflective and interactive elements.

### Professional personnel's attitudes towards self‐injurious patients

Previous research has emphasized that the personnel awareness of SIB and willingness to care for patients who injure themselves are important to the treatment process (Ramberg & Wasserman 2003). For any intervention to be successful, the importance of positive attitudes of health care personnel towards self‐injurious patient cannot be overestimated (Linehan 1999, Anderson *et al*. 2005). Of equal importance to better treatment outcomes is the emphasis on the removal of obstacles that prevent patients from seeking treatment in the first place, or adhering to it. One such significant obstacle for people who engage in SIB is stigma (Sirey *et al*. 2001). In their suicide prevention report World Health Organization (2014) outline fighting stigma as one of the ways forward in suicide prevention. Enduring stigmatization of people who engage in SIB both strengthens and is strengthened by professional attitudes to SIB, which in turn are affected by wider societal attitudes (Long *et al*. 2013).

Both assessment and follow up services may be affected by attitudes staff have towards the patients who engage in SIB (Herron *et al*. 2001, Crawford *et al*. 2003). Health care staff might be susceptible to negative stereotypes about patients who engage in self‐injury, which in turn may affect their judgment (Timson *et al*. 2012). Negative attitudes towards adult patients who engage in SIB have been noted to prevent clinicians from providing effective care to their self‐injurious patients (Pompili *et al*. 2005) and to negatively affect outcome of treatment (Arnold 1994).

Previous research has established that negative attitudes are reflected in personnel being less effective and less compassionate in their helping behavior (Mackay & Barrowclough 2005, Pompili *et al*. 2005, Saunders *et al*. 2012), and can also in themselves become a risk factor for suicide (Horvath & Symonds 1991, Morgan & Priest 1991). Earlier studies have found that health professionals’ feelings towards suicidal patients are often ambivalent and complicated (Talseth *et al*. 2001) and that mental‐health professionals are often reluctant to talk to suicidal patients (Samuelsson *et al*. 1997, Talseth *et al*. 2001), although the patients themselves view verbal contact with the personnel as essential both for the treatment process and for the desire to continue living (Samuelsson *et al*. 2000). McGaughey *et al*. (1995) argued that communication difficulties together with negative stands can reinforce the stigma associated with SIB and endanger the effectiveness of professional interventions. The high prevalence of negative attitudes among personnel caring for suicide attempters has been deemed alarming (Samuelsson *et al*. 1997).

When it was examined how patients themselves view their treatment, their reports often indicated that staff attitudes are negative, and their behavior towards patients was punitive (National Institute for Clinical Excellence (NICE) 2004). Patients and their family members further felt that they were directly stigmatized by staff, and that patients’ suicide attempt was not taken seriously by the staff (Cerel *et al*. 2006). Another study found that patients who are not met with positive and empathic attitudes are less likely to remain in Emergency Department and to engage in assessment treatment (McAllister *et al*. 2002a,b). Furthermore, according to a study by Samuelsson *et al*. (2000) lack of confirmation of the self by the health care professional has been found to leave patients with a strong desire to repeat SIB.

Studies have also indicated positive attitudes among health care staff towards patients presenting with SIB (Suokas *et al*. 2008, Conlon & O'Tuathail 2012), as well as strong desire to help such patients (Gibb *et al*. 2010). Verbal contacts with the staff were seen by patients as essential for the process of healing and for the desire to go on living (Samuelsson *et al*. 2000). Experiencing the staff as kind, respectful and nonjudgmental seemed to contribute to relief from shame in those patients (Wiklander *et al*. 2003). A respectful attitude by health care professionals towards suicidal patients has been found to ease patients’ discomfort and instill hope, as well as affirm self (Wiklander *et al*. 2003, Lindgren *et al*. 2004).

To sum up, improving attitudes of personnel towards patients who engage in SIB ought to minimize avoidance by personnel and enhance their desire to work with these patients. In addition, attitude improvement should further contribute to better treatment outcomes with respect to patients who self‐injure (Chan *et al*. 2009, Gibb *et al*. 2010).

### Improving attitudes through training

It has been suggested that staff training in working with self‐injurious patients could have the potential to increase staff attitudes and to enhance patient care (Gibb *et al*. 2010). Results have shown that training in working with self‐injurious patients may be an effective means of changing negative attitudes towards self‐injury (Samuelsson & Åsberg 2002, Ramberg & Wasserman 2003, Norheim *et al*. 2013), and can lead to improvement in the quality of psychosocial assessment of these patients (Crawford *et al*. 1998).

Others too have reported the improvements in attitudes following brief training in intervention programs (Gask *et al*. 2006, Botega *et al*. 2007, Perboell *et al*. 2015). A specialized training in how to care for patients who engage in SIB has been found to result in a more positive attitude towards these patients and a greater closeness with them (Karman *et al*. 2015). Results of previous studies have shown that more positive attitudes were associated with previous training in suicide risk assessment (Herron *et al*. 2001), and that nurses who had attended education on SIB had more positive attitudes than non‐attendees (McCann *et al*. 2007).

In addition to improving attitudes towards SIB, training can boost confidence to work with suicidal patients and can improve clinical practice skills, thus benefiting both the nurses and the patients (Jacobson *et al*. 2012, Perboell *et al*. 2015).

The lack of training available in the institutions that prepare mental health professionals has been documented for decades (Schmitz *et al*. 2012), indicating a strong need to organize more training programs on SIB prevention, so that these professionals could be better prepared to work with patients who self‐injure (Devine 1989). The absence of formal, systematic, evidence‐based training in assessing the risk and treating these patients is contributing to the low‐level skills among health care personnel (Jacobson *et al*. 2012). Staff feel inadequately trained to care for self‐injurious patients and would welcome the training in this area (Gibb *et al*. 2010). In addition to improving the training, there is a need to implement strategies that would enhance working alliance and inform practice (Conlon & O'Tuathail 2012). Because psychiatric personnel frequently have to deal with self‐injurious patients in emergency situations, with their own skills only to rely upon, they need specific training in assessment and treatment of SIB.

To sum up, the need for training has been recognized in previous research into the improvement of attitudes of personnel towards self‐injurious patients (Ramberg & Wasserman 2003, Artis & Smith 2013).

### Conceptualizing attitudes and attitude change process

To understand the processes behind attitude expression and attitude‐change in staff caring for patients who self‐injure this study adopts Devine's (1989) definition of attitudes as acceptance of the content of a cultural stereotype. Stereotypes and attitudes are considered as distinct cognitive structures, each of which represents a part of a person's complete base of knowledge about a particular group. Stereotypes are instituted in children's memories before the development of the cognitive flexibility to critically assess the validity of a stereotype. Attitudes – propositions that are accepted as being true – however are newer cognitive structures. They can differ from what one knows about a particular group and from the affective reactions towards that group. According to Devine and Elliot (1995), stereotypes and attitudes are activated by different cognitive processes. Stereotypes, due to having a long socialization history and having been frequently activated, can be activated automatically, providing a ‘default’ basis for responding when presented with the attitude object. The implication here is that the ‘default’ response is inevitably a stereotype‐based response. The ‘default’ response to people who engage in self‐injury was well described by Walsh and Rosen (1988) more than 20 years ago ‘..we inevitably experience discomfort when encountering fellow human beings so intensely distressed that they cause themselves concrete physical harm’ (p.3).

Because the attitudes are not as easily accessible as stereotypes, the overt non‐prejudiced response involves controlled cognitive processes. These processes in turn enable both the intentional inhibition of the automatically activated stereotype and the activation of an attitude. According to Devine and Monteith (1993), people can inhibit stereotype‐based response only if they have the *time* and the cognitive *capacity* to set controlled process into motion, in order for it to bring the personal attitudes to awareness. According to this model the knowledge and information that is activated in the stereotype can influence information processing to follow. Thus, the behavior of those whose attitudes do not accept the stereotype can nonetheless be influenced by its activation provided they do not consciously observe this activation. Furthermore, this model posits that both stereotypical thoughts and non‐prejudiced attitudes can co‐exist within the same individual. The change from prejudice to non‐prejudice is not seen as an all‐or‐none event, but rather as a process.

This change has been studied in terms of conditions under which people are persuaded by others. Within this context the most frequently promoted concept was that of involvement (Hovland *et al*. 1957). Johnson and Eagly (1989) specified that different types of involvement exist and they have different effects on persuasion. This study assumes that the type of involvement relevant to the persuasion of psychiatric personnel and their attitude change is outcome‐relevant involvement. According to Johnson and Eagly (1989) outcome‐relevant involvement is the extent to which the attitudinal matter under elaboration is personally significant to individuals’ present important goals or outcomes. The same theory posits that with outcome‐relevant involvement, high‐involvement individuals are more persuaded by strong arguments and less by weak arguments. Those who are less involved however, tend to favor weaker arguments.

To sum up, improving psychiatric personnel's attitudes entails learning how to inhibit stereotype‐based responses and replace them with personal attitude responses. This attitude change process is likely aided by strong argument persuasion of involved subjects. Thus, to facilitate attitudinal change, the training employed in this service is to be based on strong evidence‐based arguments, supported by practical clinical examples, and is in its nature reflective and interactive.

## Aim

This study examines attitudes towards self‐injurious patients among healthcare staff at the psychiatric clinic. It evaluates the effect of a structured clinical training program in evidence‐based SIB assessment and treatment on psychiatric personnel's attitudes towards patients who self‐injure.

The study's aims were following: (1) to describe the attitudes among psychiatric personnel (especially understanding view of the psychiatric personnel and willingness to care) towards patients who have self‐injured; (2) to detect whether age, education, frequency of self‐injurious patients contact, and work experience of the personnel are associated with existing attitudes, and (3) to detect whether the structured clinical training program in evidence‐based SIB assessment and treatment has positive impact on psychiatric personnel's attitudes towards patients who self‐injure.

## Method

This study took place in North Karelia Central Hospital Psychiatric Clinic in Finland. The hospital's catchment area comprises the North Karelia district (165 445 inhabitants in 2013). The study was conducted in 2014. The first measurement took place in January 2014, prior to the training. The second measurement took place in June 2014 post training. Participating staff were asked to complete the Understanding Suicidal Patients Questionnaire – USP (Samuelsson *et al*. 1997) before and after the training.

### Participants

The participants of this study were the 50 staff members of the North Karelia Central Hospital Psychiatric Clinic who took part in the Suicide and Self Harm Assessment and Treatment Training. The participants were a multi‐professional group, reflecting clinical practice in Finland, where those who engage in SIB are often referred to a variety of different professionals, such as nurses, psychiatrists and psychologists. Internationally too, it has been noted that SIB should be considered as a multi‐professional issue (Turp 1999, Timson *et al*. 2012).

Participation in the training was voluntary but the places were limited to 50 participants. The design and the aims of the study were explained to participants both face‐to‐face and in a letter. All 50 participants returned the questionnaire. Table 1 presents the background of participants in further detail.

**Table 1:** Background of the participants

	*N* (%)
Sex	
Male	8 (16)
Female	42 (84)
Age	
<25 years	0 (0)
25‐40 years	19 (38)
>40 years	31 (62)
Education	
Nurse	23 (46)
Specialist nurse	11 (22)
Doctor	1 (2)
Specialist Doctor	4 (8)
Mental Health Nurse	8 (16)
Psychologist	3 (6)
Frequency of suicidal patients contact	
Daily	10 (20)
Weekly	11 (22)
Monthly	14 (28)
Seldom	15 (30)
Work experience	
Less than 5 years	11 (23)
6‐15 years	17 (34)
16‐25 years	10 (20)
Over 26 years	12 (23)

### The Training Program

The content of the training was developed from the framework provided by Worchel and Gearing's (2010) Evidence‐Based Suicide Assessment and Treatment. This framework was expanded by consulting the World Health Organization protocol for suicide prevention, recent research into assessment and treatment of suicidal and non‐suicidal self‐injurious behavior, and specific Finnish institutional policies. Once developed, the training program was reviewed and validated by an expert panel of professionals including two consultant psychiatrists, a mental health nurse, a psychologist and a psychologist academic. The panel deemed the program content to be accurate and compatible with the standards generally accepted by medical science. The training program was provided as continuing professional development.

The training contained strong reflective and interactive elements, as these were identified to be of particular benefit in education that aims at attitude change amongst staff towards self‐injury (Karman *et al*. 2015). The training adopts Chan *et al*. (2009) definition of reflective learning as the process ‘of internally examining and exploring an issue of concern triggered by an experience, which creates and clarifies meaning in terms of self and results in a changed conceptual perspective’ (p.764). An example of activity that leads to reflective learning is engaging in dialogue and thereby improving one's ability to form perspectives. Case discussions and role‐play result in more structured dialogue which renders itself to linking theory and practice. According to Branch and Paranjape (2002), reflection can result in deepened self‐awareness, including awareness of personal values and attitudes.

The training was delivered over 4‐month period, with frequency of one full day per month. The trainer was the first author, who is a psychologist specialized in psychotherapy training. Table 2 presents the training program in more detail.

**Table 2:** Content of the training

Day 1	Day 2
Defining SIB	Assessment of SIB
Ethical and legal issues in suicide and self‐injury	Fundamental SIB Assessment components
Professional ethics	Sociodemographic data
SIB and the law	Symptom history
Communication and building a positive relationship	Current SIB SIB history
Verbal and non‐verbal communication	Family SIB history
Validation	Risk factors
Hope and optimism	Protective Factors
Positive view of future	SIB Rating Scales: Columbia, SASII
Role playing to practice building positive relationship	Intervention planning based on the assessment SIB and mental illness
Reflective elaboration	Reflective elaboration Homework: Apply rating scales at work

Day 3	Day 4
Crisis Intervention and SIB	Cognitive‐Behavioral Therapy and SIB
The definition and the key factors of crisis	The theory behind CBT
Robert's Seven‐Stage Crisis Intervention Model	The major components of CBT
Core Stages in crisis intervention	Core strategies used in CBT
Dialectic Behavioral Therapy and SIB	SFBT + ACT 4 session model and SIB
The theory behind DBT	Small group exercise: Reading transcript of session where CBT was employed
The major components of DBT	
Core strategies used in DBT	
Role play to practice intervention strategies Reflective elaboration	Evaluation of the course

### Instrument

The Understanding Suicidal Patient (USP) Questionnaire was used. This questionnaire was developed from a questionnaire by Suokas and Lönnqvist (1989) and modified by Samuelsson *et al*. (1997). The 11 items were summed to form the USP Scale, which is intended to measure understanding and willingness to care for patients who have attempted suicide. A higher score on this scale indicates less favorable attitude towards self‐injurious patients. In earlier studies (Samuelsson *et al*. 1997, Samuelsson & Åsberg 2002) the scale has demonstrated satisfactory internal consistency and good reliability; the mean inter‐item correlation for the scale in those studies was 0·20, and Cronbach's α was 0·74. Each statement was scored on a 5‐point Likert scale ranging from ‘I agree completely’ ‐ ‘I disagree completely’. All USP statements are presented in Table 3. Both prior to and after the training the participants were presented with the USP forms, which they answered anonymously.

**Table 3:** Comparison of items in USP Pre (*n* = 50) and Post (*n* = 50) training

	Mean(sd) Pre	Mean(sd) Post	*t* (d.f.)	*P* values
1. Patients who have tried to commit suicide are usually treated well in my work unit	1·98 (1·22)	1·08 (0·27)	5·08 (98)	0·000
2. I sometimes show my irritation with a patient who has tried to commit suicide^a^	3·88 (1·22)	4·22 (1·05)	−1·49 (98)	0·140
3. A person who has made several suicide attempts is at great risk of committing suicide	1·82 (1·04)	1·28 (0·572)	3·21 (98)	0·002
4. I nurse patients who have tried to commit suicide as willingly and sympathetically as I nurse other patients	1·80 (0·93)	1·54 (0·73)	1·56 (98)	0·123
5. Because the patients who have tried to commit suicide have emotional problems. they need the best possible treatment	1·48 (0·95)	1·08 (0·27)	2·85 (98)	0·005
6. I often find it difficult to understand a person who has tried to commit suicide^a^	3·82 (1·02)	4·02 (0·93)	−1·02 (98)	0·311
7. I like to help a person who has tried to commit suicide	1·8 (1·05)	1·72 (0·95)	0·40 (98)	0·690
8. I try to do my best to talk with a patient who has attempted suicide about his or her personal problems	1·54 (1·01)	1·54 (0·84)	0·00 (98)	1·00
9. It is usually troublesome to nurse a patient who has tried to commit suicide^a^	2·78 (0·97)	2·90 (0·91)	−64 (98)	0·526
10. I am usually sympathetic and understanding towards a patient who has tried to commit suicide	2·08 (0·94)	2·06 (0·68)	0·121 (98)	0·904
11. I try to do my best to make a patient who has tried to commit suicide feel comfortable and secure	1·62 (1·01)	1·50 (0·58)	0·73 (98)	0·467

Note meaning of the scores: 1 =  totally agree, 2 =  agree, 3 =  neutral, 4 =  disagree, 5 =  totally disagree.

a Reverse scoring.

### The Training Evaluation

The participants’ training experiences were evaluated upon the completion of the training. Following the training, the participants were asked one open‐ended question using a paper‐pencil form: ‘What did you find most useful in the training?’. In the same form participants were encouraged to suggest how they would improve the training.

### Ethical considerations

The participants received written information about the study purpose and procedure, the voluntary nature of participation and were assured of complete confidentiality. All participants gave informed and written consent to participate in the study, which was approved by the chief physician of the psychiatric policlinic. Data were collected anonymously. Since there were no patients involved in the research, review and approval by the Ethics Committee was not necessary according to Finnish legislation.

### Data analysis

To ensure reliability of the scale, the 11 USP items were subjected to an item analysis. The mean inter‐item correlation for the scale was 0·33, and Cronbach's α was 0·85. Based on the 25th and 75th percentiles, a positive attitude PreTraining was defined as a USP scale score under 21 and a negative attitude as a score of 26 or over; a positive attitude PostTraining was defined as a USP scale score under 21 and a negative attitude as a score of 25 or over.

The mean differences before and after the training were tested by unpaired *t*‐test. To keep anonymity as strict as possible, the individual answers before and after the training could not be identified. Therefore, a paired *t*‐test could not be used. To test the differences in proportions on single items, the two‐tailed tests of significance were employed, and *P* values of <0·05 were considered to be statistically significant. Non‐parametric methods (the Mann–Whitney *U*‐test and Kruskal–Wallis test) were also used in parallel (data not shown, as the results were very similar to those obtained with the parametric methods). Two‐way anova was used to test impact of background variables on the USP scores.

For any statistically significant finding within group effect size (ES) using a Cohen's *d* was calculated to determine the magnitude of the change (in purpose to estimate the clinical significance of the change). The within‐group ES was calculated by dividing the mean change from pre‐to post‐ measurement by the combined (pooled) standard deviation (sd) of the two measurements (Cohen^68^; *d* = M1‐M2/sd). A *within‐group* ES of 0·2 was considered small, 0·5 medium and 0·8 large. Qualitative data regarding the participants’ evaluation of the useful aspects of the training were analyzed using thematic analysis as described by Braun and Clarke (2006).

## Results

### Primary outcomes

The mean score for the whole USP Scale PreTraining was 24·60 ± 5·40 (range 19·00‐46·00; 25% percentile 21·00, median 23·00, 75% percentile 26·00), and the mean score for the whole USP Scale PostTraining was 22·94 ± 2·72 (range 19·00‐30·00; 25% percentile 21·00, median 22·50, 75% percentile 25·50), *t* = 1·94, d.f. = 98, *P* = 0·055, *d* = 0·39.

Lower scores signify more understanding responses, i.e. a more favorable attitude. The distribution of the scores on most items tended to be somewhat skewed toward the ‘more understanding’ end of the scale. Pre‐ and PostTraining differences in individual items of the USP Scale are presented in Table 3.

The training program had statistically significant impact (*P* < 0·01) on the following individual items of the USP scale (the within group effect size varied from medium *d* ≥ 0·50, to large *d* ≥ 0·80): patients who have tried to commit suicide are usually treated well in my work unit (*d* = 1·02; item number 1); a person who has made several suicide attempt is at greater risk of committing suicide (*d* = 0·64; item number 3); because the patients who have tried to commit suicide have emotional problems, they need the best possible treatment (*d* = 0·57; item number 5). Thus, the training had a positive impact on personal's attitudes on these items, and the change (based on effect size) was medium or large.

**Figure 1:**
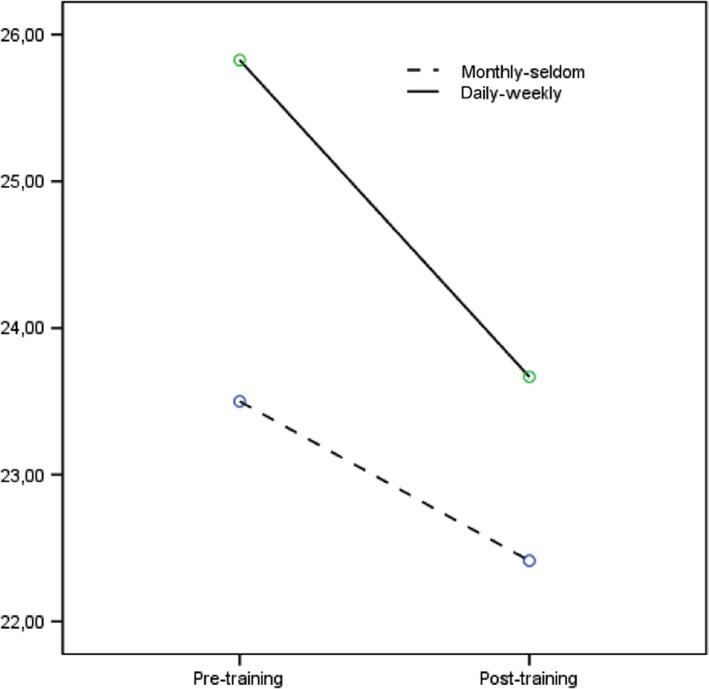
Frequency of contact vs. USP score Pre and PostTraining

When the impact of background variables on the total score of USP was investigated, the results of two‐way anova revealed statistically significant main effect for the frequency of contact, *F*(1) = 4·34, *P* = 0·04, ɳ^2^_p _= 0·044, as presented in Figure 1. This suggested that psychiatric personal having a more frequent contact with the self‐injure patients (daily or weekly) reported higher USP scores (reflecting a more negative attitude) as compared to those having a seldom contact with these patients (monthly or more seldom). However, the between group effect size indicated a small overall difference between the groups (*d* = 0·42; total USP score m = 24·80, sd 5·66 for the daily/weekly group, and m = 22·93, sd 2·71 for the seldom contact group).

On further analyses, it was observed that the training had significant impact on Items 1 and 5 among psychiatric personal having frequent (daily/weekly) contact with the patients. The scores on Item 1 (‘Patients who have tried to commit suicide are treated well in my work unit’) changed significantly more to the positive direction among those having frequent contact with the patients (*F*(1,95) = 12·21, *P* = 0·001; Daily/weekly: pre, m = 2·61, sd 1·44, post, m = 1·10, sd 0·30; Seldom: pre, m = 1·46, sd 0·65; post, m = 1·07, sd 0·26). Also, the scores on Item 5 (‘Because the patients who have tried to commit suicide have emotional problems, they need the best possible treatment’) changed significantly more to the positive direction among those having frequent contact with the patients (*F*(1,95) = 8·59, *P* = 0·004; Daily/weekly: pre, m = 1·83, sd 1·23, post, m = 1·05, sd 0·22; Seldom: pre, m = 1·12, sd 0·33; post, m = 1·10, sd 0·31). Age, profession and length of work experience, did not correlate with specific items of USP PostTraining.

### Evaluation of the training

All 50 participants answered the paper‐pencil question about the most useful aspects of the training. The following themes emerged as the most useful aspects of the training: (1) tools to use in the field: concrete and structured; (2) knowledge base: broad, up‐to‐date, and evidence‐based; (3) trainer: professional, and inspirational; (4) group exercises and (5) subject: highly relevant. The themes that emerged as suggestion to improve the training were: (1) more training days; (2) more case examples; (3) more intervention detail.

## Discussion

The results of this study find that: (1) participants’ attitudes towards self‐injurious patients as measured by USP PreTraining were neutral; (2) the frequency of contact with self‐injurious patients was associated with the existing attitudes and (3) attitudes as measured by USP improved immediately after completing the training.

The finding that the attitudes of psychiatric personnel were neither entirely positive nor entirely negative, are in agreement with Devine and Elliot's (1995) attitude theory which cautions against viewing attitudes and their change as all‐or‐none event, and posits that positive and negative attitudes can coexist within the same individual (Devine *et al*. 1991). The present study sees the ‘neutrality of the attitudes’ result as an expression of participants’ reality, where existing attitudes are mixed and complex, and thus cannot neatly fall in either all negative or all positive categories. Previous studies too have found staff attitudes towards this group of patients to be ambivalent and complicated (Palmer 1993).

The finding that at the outset the attitudes of those who frequently encounter patients engaging in SIB were less favorable when compared to the attitudes of those who encounter this group of patients less frequently warrants further discussion. As noted earlier, previous research suggests that there exists a very strong desire amongst health care staff to help patients who self‐injure (Gibb *et al*. 2010). However, due to the nature of SIB as repetitive behavior, frequently encountering self‐injurious patients may lead to feelings of frustration in personnel, as they may see themselves as unable to achieve their goal of ‘curing’ these patients. The frustration may also arise from the personnel seeing that these patients are not receiving the type of care they need. Staff that do not encounter these patients frequently are accordingly spared this frustration or feeling of failure, and this could account for their initial more favorable attitudes – it is likely to reflect portrayal of their working reality, which by being in lesser contact with of this group of patients, is also less privy of the mixed emotions related to their assessment and treatment.

In addition to emergency service, the first line of contact for patients who self‐injure is psychiatric care. Both these contexts are known for their fast pace of work, time pressure, pressure for results and the consequent work stress (Suokas & Lönnqvist 1989). Under time pressure, automatic cognitive processing is activated, resulting in stereotypical, ‘default’ response, which is seen as ‘less favorable’ attitude. It is important here to bring to attention the distinction between stereotype and attitude. Attitudes of the staff frequently encountering SIB patients may well in reality be more favorable, yet, under the given constraints they are overshadowed by more easily available stereotypical responding. On the other hand, for those less frequently encountering these patients, in the context where working pace is less quick, there is time to bring controlled cognitive processes into action and thus inhibit intentionally the automatically activated stereotype, thus resulting in expression of personal attitude, reflected here as ‘more favorable’.

Finally, the third major finding of current study was the improvement in attitudes immediately following the training. In understanding this finding, the concept of outcome related involvement is essential. According to cognitively oriented persuasion researchers, it is the involvement, in this case enhanced by the training program, that increases motivation of the participants to process or engage in information discussed during the training. The more frequent the involvement then, the greater the information processing and application of material presented in the training. The findings from the qualitative data of this study regarding the most useful aspects of the training are in agreement with previous findings that with outcome‐relevant involvement, high‐involvement subjects are more persuaded by strong arguments. Strong arguments can be detected in the themes ‘knowledge base: evidence‐based’ and ‘trainer: professional’. The likely persuasion of highly involved participants by these strong evidence‐based arguments is reflected in changes on the items ‘Because the patients who have tried to commit suicide have emotional problems, they need best possible treatment’ and ‘I sometimes show my irritation with a patient who has tried to commit suicide’. Both the complexity of these patients’ needs and the negative consequences of staff showing their irritation were discussed during the training in the light of evidence‐based research. Elsewhere too, it was found that greater and more accurate knowledge of contextual issues can de‐stigmatize SIB by providing health care personnel with alternative explanations for the behavior, which in turn can increase empathic attitudes and lead to changed behavioral responses (Crawford *et al*. 2003, Anderson *et al*. 2005).

The theme ‘concrete tools to use in the field’ on the other hand is likely to reflect both the higher need of highly involved staff for these tools, as well as their greater opportunity to apply these during the training, as opposed to staff with less frequent involvement with these patients. Higher involvement, using tools deemed helpful can help better understand the change in the item ‘Patients who have tried to commit suicide are usually treated well in my work unit’. Horowitz *et al*. (2001) reported that having a screening tool is not only preferred by nurses but can also increase clinicians’ confidence. The qualitative data also revealed the theme of ‘more training days’ as means of improvement of the training. The same variable, further professional training in the same subject, may also have had effect on the improvement of attitudes amongt highly involved, as it was in previous research too associated with reduced stress in professionals and better care for self‐harming patients (Crawford *et al*. 2003).

While availability of supervision, a standard in Finnish health care, may have positive impact on the attitudes in general, an improvement following the training implies that supervision should be supplemented with specific training in SIB – a finding raised in previous research (Ramberg & Wasserman 2003). The type of training applied in this study, where the staff were permitted to examine their own values and beliefs in a secure environment, and with a professional trainer, has been deemed especially beneficial in attitudinal change (Burrow 1994).

The type of training applied in this study has positive impact on the attitudes of personnel who frequently works with SIB patients, and should because of this in the future be offered to this group of health care staff. The positive impact is most evident in endorsement of beliefs that patients who self‐injure are treated well and that these patients need best possible treatment.

### Practice considerations

To improve attitudes towards, and ensure the quality of treatment of patients who self‐injure, the service‐providers need to train their personnel in evidence‐based assessment and treatment of these patients. It is especially important to provide personnel who encounter this group of patients with educational opportunity to examine, in a reflective learning environment, and in non‐judgmental way, their own attitudes towards this patient group and ways of how attitudes can influence behavior. In particular, personnel should be encouraged to reflectively examine their own negative emotional reactions to these patients, and should be educated as to how expressed negative emotion affects treatment outcome. The personnel need to be provided with an opportunity to practice using structured and psychometrically sound assessment instruments, as well as setting treatment goals that are realistic and achievable. When working under time constraints personnel need to set strict boundaries on high‐risk case load.

## Limitations

This study should be regarded as a pilot study, because of the limited number of participants. As the questionnaire method was used, there remains a possibility that social desirability has influenced the results. Attempt was made to reduce this possibility through anonymity. In addition, questionnaire studies are a useful baseline measures in intervention studies that investigate the effectiveness of projects aiming at changes in attitudes (McAllister *et al*. 2002a,b). A definite strength in this study is 100% questionnaire response rate. This study should however be regarded as a pilot study, because of the limited number of participants.

Some misunderstanding may result from using the USP to study the attitudes towards all self‐injurious patients, suicidal and non‐suicidal. However, as previously indicated, clinical practice in Finland at present moment does not make this distinction, but rather classifies all self‐injurious patients under ‘attempted suicide’ diagnostic, thus making the use of USP justifiable.

Another limitation is that the study does not provide knowledge of the potential changes in attitudes that may have occurred in the same period among personnel who did not attend the training. Thus, the study cannot rule out alternative explanations for the change in attitudes observed. A controlled replication study would be needed to resolve these concerns.

Although the study shows change in attitudes following the training, it is not known if this can result in changes in behavior as a result of the training. It is however known from previous research that relationship exists between attitudes and practice behaviors (Jacobson *et al*. 2012) and that attitudes toward SIB have a direct impact on the relationship staff have with these patients and the quality of care they provide (McDonough *et al*. 2004).

## Author contribution

Taking into account the instructions given and comments made by the co‐authors (supervisors), the first (corresponding) author collected the data, conducted the analyses and wrote the manuscript.

All authors have agreed on the final version and meet at least one of the following criteria [recommended by the ICMJE (http://www.icmje.org/recommendations/)]:

- substantial contributions to conception and design, acquisition of data, or analysis and interpretation of data;
- drafting the article or revising it critically for important intellectual content.
